# Supplementary material for: Multi-City Analysis of the Acute Effect of Polish Smog on Cause-Specific Mortality (EP-PARTICLES Study)
Source: Int J Environ Res Public Health. 2023 Apr 18;20(8):5566. doi: 10.3390/ijerph20085566 (PMC10139136; doi:10.3390/ijerph20085566)
Supplement: Supplementary file 1 [file ijerph-20-05566-s001.zip › ijerph-2195395-supplementary.pdf]

Table S1. Association between weather conditions and occurrence of ACS- and IS-related deaths. Results are presented as ORs with 95% CIs using CLR.

Mortality	LAG	Group	Mean temperature			Mean humidity			Mean atmospheric pressure (sea level)		
			OR	95% CI	p	OR	95% CI	p	OR	95% CI	p
ACS	LAG 0	Overall population	1.021	0.956-1.089	0.54	0.986	0.962-1.011	0.28	0.981	0.951-1.011	0.21
		Male	1.03	0.94-1.128	0.53	0.972	0.939-1.006	0.11	0.982	0.94-1.025	0.4
		Female	1.012	0.923-1.109	0.81	1.002	0.966-1.038	0.93	0.98	0.937-1.024	0.36
		Over 65 years old	1.029	0.959-1.104	0.43	0.994	0.967-1.021	0.65	0.981	0.949-1.014	0.25
		Under 65 years old	0.971	0.819-1.151	0.73	0.945	0.885-1.008	0.08	0.979	0.902-1.063	0.62
	LAG 1	Overall population	0.986	0.923-1.054	0.68	0.998	0.973-1.024	0.88	0.985	0.955-1.016	0.35
		Male	0.971	0.885-1.066	0.54	0.988	0.954-1.024	0.52	1	0.957-1.045	0.99
		Female	1.002	0.913-1.101	0.96	1.009	0.972-1.046	0.65	0.971	0.929-1.015	0.19
		Over 65 years old	0.986	0.918-1.059	0.7	1.007	0.98-1.035	0.61	0.986	0.953-1.02	0.41
		Under 65 years old	0.983	0.826-1.17	0.85	0.947	0.886-1.011	0.1	0.979	0.902-1.063	0.61
	LAG 2	Overall population	0.978	0.917-1.043	0.49	0.991	0.966-1.016	0.47	0.989	0.963-1.015	0.4
		Male	0.974	0.889-1.066	0.57	0.984	0.949-1.02	0.37	0.994	0.959-1.031	0.76
		Female	0.982	0.896-1.076	0.69	0.998	0.962-1.035	0.92	0.983	0.947-1.02	0.37
		Over 65 years old	0.975	0.909-1.045	0.47	0.994	0.967-1.022	0.67	0.984	0.957-1.012	0.26
		Under 65 years old	0.994	0.838-1.179	0.95	0.971	0.907-1.04	0.4	1.017	0.95-1.09	0.62
IS	LAG 0	Overall population	1.021	0.956-1.089	0.54	0.986	0.962-1.011	0.28	0.981	0.951-1.011	0.21
		Male	1.03	0.94-1.128	0.53	0.972	0.939-1.006	0.11	0.982	0.94-1.025	0.4
		Female	1.012	0.923-1.109	0.81	1.002	0.966-1.038	0.93	0.98	0.937-1.024	0.36
		Over 65 years old	1.029	0.959-1.104	0.43	0.994	0.967-1.021	0.65	0.981	0.949-1.014	0.25
		Under 65 years old	0.971	0.819-1.151	0.73	0.945	0.885-1.008	0.08	0.979	0.902-1.063	0.62
	LAG 1	Overall population	0.986	0.923-1.054	0.68	0.998	0.973-1.024	0.88	0.985	0.955-1.016	0.35
		Male	0.971	0.885-1.066	0.54	0.988	0.954-1.024	0.52	1	0.957-1.045	0.99
		Female	1.002	0.913-1.101	0.96	1.009	0.972-1.046	0.65	0.971	0.929-1.015	0.19
		Over 65 years old	0.986	0.918-1.059	0.7	1.007	0.98-1.035	0.61	0.986	0.953-1.02	0.41
		Under 65 years old	0.983	0.826-1.17	0.85	0.947	0.886-1.011	0.1	0.979	0.902-1.063	0.61
	LAG 2	Overall population	0.978	0.917-1.043	0.49	0.991	0.966-1.016	0.47	0.989	0.963-1.015	0.4
		Male	0.974	0.889-1.066	0.57	0.984	0.949-1.02	0.37	0.994	0.959-1.031	0.76
		Female	0.982	0.896-1.076	0.69	0.998	0.962-1.035	0.92	0.983	0.947-1.02	0.37
		Over 65 years old	0.975	0.909-1.045	0.47	0.994	0.967-1.022	0.67	0.984	0.957-1.012	0.26
		Under 65 years old	0.994	0.838-1.179	0.95	0.971	0.907-1.04	0.4	1.017	0.95-1.09	0.62

Abbreviations: ACS, acute coronary syndrome; CI, confidence interval; CLR, conditional logistic regression; IS, ischemic stroke; OR; odds ratio; p, p-value
